# One-Year Effect of Elexacaftor/Tezacaftor/Ivacaftor Therapy on HbA1c Levels and Insulin Requirement in Patients with Insulin-Dependent Cystic Fibrosis-Related Diabetes: A Retrospective Observational Study

**DOI:** 10.3390/life14101309

**Published:** 2024-10-16

**Authors:** Marta Bassi, Marina Francesca Strati, Gaia Spiandorello, Marta Scalas, Federico Cresta, Maria Grazia Calevo, Giuseppe d’Annunzio, Carlo Castellani, Nicola Minuto, Mohamad Maghnie, Rosaria Casciaro

**Affiliations:** 1 Department of Neuroscience, Rehabilitation, Ophthalmology, Genetics, Maternal and Child Health (DINOGMI), University of Genoa, 16147 Genoa, Italy; martabassi@gaslini.org (M.B.); marinastrati29@gmail.com (M.F.S.); spiandorellogaia@gmail.com (G.S.); martascalas@yahoo.it (M.S.); mohamadmaghnie@gaslini.org (M.M.); 2 Epidemiology and Biostatistics, Scientific Direction, IRCCS Istituto Giannina Gaslini, 16147 Genoa, Italy; mariagraziacalevo@gaslini.org; 3 Regional Cystic Fibrosis Centre, IRCCS Istituto Giannina Gaslini, 16147 Genoa, Italy; federicocresta@gaslini.org (F.C.); carlocastellani@gaslini.org (C.C.); rosariacasciaro@gaslini.org (R.C.); 4 Pediatric Clinic, IRCCS Istituto Giannina Gaslini, 16147 Genoa, Italy; giuseppedannunzio@gaslini.org

**Keywords:** CFRD, cystic fibrosis, diabetes, CFTR modulators, ETI treatment

## Abstract

Introduction: The impact of ETI therapy on pulmonary function and nutritional status has been widely studied; the literature on the possible outcomes on glycemic control and insulin requirement in patients affected by CFRD is controversial. Aim: The main objective of our study was to evaluate HbA1c levels in patients with cystic fibrosis-related diabetes (CFRD) after one year of therapy with elexacaftor/tezacaftor/ivacaftor (ETI). The secondary objective was to study the changes in the total daily insulin dose (TDD), pulmonary function and metabolism in this population. Materials and methods: A retrospective single-center observational study was conducted at the Regional Cystic Fibrosis Centre and Diabetology Centre of IRCCS Istituto Giannina Gaslini. The observation period was divided into four different time points: initiation (T0), 3 months (T_3mo_), 6 months (T_6mo_) and 12 months (T_12mo_) of ETI therapy. Demographic and clinical data were collected. The results were then stratified by genotype (homozygous or heterozygous F508del). Results: Twenty-eight patients with CFRD undergoing insulin therapy were included. TDD (IU) significantly decreased at T_3mo_ and T_6mo_, but not at T_12mo_, whereas HbA1c decreased significantly at all three times. The number of hospitalizations and pulmonary exacerbations decreased significantly. Conclusion: We demonstrated both improvement in glycemic control (by means of HbA1c) and insulin requirement in insulin-dependent CFRD patients after one year of ETI treatment.

## 1. Introduction

Cystic fibrosis-related diabetes (CFRD) is a specific extra-pulmonary complication associated with increased morbidity and mortality in the cystic fibrosis (CF) population. Among the extrapulmonary complications of patients with CF, CFRD is by far the most common [1]. CFRD is a unique form of diabetes, most frequently diagnosed in young adults and the prevalence increases with age [2]. The etiology of CFRD seems to be linked to the partial loss or dysfunction of pancreatic islets, leading to insulin deficiency and to insulin resistance [3]. The etiology of insulin secretion impairment is also linked to a direct effect of the CF transmembrane conductance regulator (CFTR) protein [4]. A decline in the patient’s pulmonary function and nutritional status begins about 2–4 years prior to the diagnosis of CFRD [5]. This deterioration is believed to be associated with protein catabolism due to insulin insufficiency; the worsening of pulmonary function is directly associated with the severity of insulin insufficiency [6]. As mentioned above, CFRD is strongly associated with a decline in pulmonary function, increased frequency of exacerbations and poor nutritional status, resulting in a worse prognosis for the patient [7,8].

CFTR modulators, a group of innovative drugs used in the treatment of CF, have revolutionized the prognosis of these patients; their role is to correct and potentiate defects in the channel function [9]. Various combinations of these modulators are available; the most recent formulation available is a triple combination therapy of elexacaftor/tezacaftor/ivacaftor (ETI) approved for patients with at least one copy of the F508del variation [10]. The impact of ETI therapy on pulmonary function and nutritional status has been widely studied and confirmed, while only a few studies have been published regarding the possible outcomes on glycemic control and insulin requirement in patients affected by CFRD.

Grancini et al. demonstrated a significant reduction in glycated hemoglobin A1c (HbA1c) values after 6 months of ETI therapy but unchanged insulin requirements [9]. In a large Danish study including 321 patients with CF, a HbA1c decline was reported over 12 months of ETI treatment, which was not associated with a change in insulin usage in the sub-cohort of 26 patients with CFRD [11]. Lurquin et al. showed a significant decrease in insulin requirement during follow-up in 17 patients undergoing CFTR modulator therapy with either ETI or tezacaftor/ivacaftor, despite an increase in HbA1c levels [12]. Recently, Cohen et al. demonstrated a decrease of 120 min in the Oral Glucose Tolerance Test (OGTT) value after the initiation of CFTR modulator therapy [13].

As stated by Salazar-Barragan et al.’s systematic review, most studies on this topic used small sample sizes, and therefore their power to demonstrate the effectiveness of ETI was limited [14]. Our goal was to evaluate whether ETI is effective for glycemic control and improvement in the total daily dose of insulin in a larger cohort of patients with CFRD all requiring insulin treatment.

The primary aim was to study how HbA1c levels change in adult and pediatric patients affected by cystic fibrosis-related diabetes after one year of elexacaftor/tezacaftor/ivacaftor therapy. The secondary aim was to study the improvement of total daily insulin dose, pulmonary function and metabolism in this population.

## 2. Materials and Methods

A retrospective single-center observational study was conducted at the Regional Cystic Fibrosis Centre and the Regional Diabetology Centre of IRCCS Istituto Giannina Gaslini. All patients with insulin-dependent CFRD who started ETI therapy were included. Patients who started insulin therapy after initiating ETI were excluded. The observation period was divided into four different time points: initiation (T0), 3 months (T_3mo_), 6 months (T_6mo_) and 12 months (T_12mo_) of ETI therapy. The number of pulmonary exacerbations and hospitalizations, during the year before initiating ETI, was recorded, as well as previous use of CFTR modulators, six-minute walking test (6MWT) and chloride sweat test data during the 12 months prior to ETI initiation (pre-T0). Demographic (age, sex, ethnicity) and clinical data were collected. CFTR genotype, age of CFRD diagnosis, insulin therapy duration, age of ETI initiation and insulin regimen were collected at T0. At T0, T_3mo_, T_6mo_ and T_12mo,_ we compared weight, body mass index (BMI), forced expiratory volume in the first second (FEV1), forced vital capacity (FVC), total daily dose of insulin (TDD), basal dose of insulin, bolus dose of insulin, TDD per kilogram and glycated hemoglobin (HbA1c) values. At T0 and T_12mo,_ we compared the results of the cystic fibrosis questionnaire (CFQ-R) that measures the health quality of life of patients with CF (the higher the score, the better the mental status). At pre-T0 and T_6mo,_ we compared 6MWT and chloride sweat test data; and at pre-T0 and T_12mo,_ we compared the number of pulmonary exacerbations and hospitalizations. The same data were consequently subanalyzed according to the different CFTR genotypes (Group 1—subjects F508del/F508del vs. Group 2—subjects F508del/any other CF-causing mutation).

### Statistical Methods

Data are described as mean and standard deviation (SD) or median and range for continuous variables, and as absolute and relative frequencies for categorical variables. Comparisons between T0, T_3mo_, T_6mo_ and T_12mo_ to examine continuous variables were performed using the paired Wilcoxon test. *p* values ≤ 0.05 were considered statistically significant, and all *p* values were based on two-tailed tests. Statistical analysis was performed using SPSS for Windows (SPSS 29-2022, Sept. SPSS Inc., Chicago, IL, USA). Data were extracted from electronic medical records.

This study was conducted in accordance with the Declaration of Helsinki. According to Italian legislation, the study did not need ethical approval, as it was a purely observational retrospective study on twenty-eight patients with anonymously collected data. Furthermore, it was not possible to request informed consent for participation in the study, given the nature of the study. In any case, consent to completely anonymous use of clinical data for research/epidemiological purposes was requested by the clinical team at the time of admission/diagnostic procedure.

## 3. Results

In total, 28 Caucasian patients (16 males vs. 12 females; 57.1% vs. 42.9%) affected by CF and diagnosed with CFRD undergoing insulin therapy followed at our regional CF centre were included, with a median age of 33 years (SD 13.26, minimum age 12 years, maximum age 56 years) at the start of ETI therapy. Only one patient was excluded due to the initiation of insulin therapy after beginning ETI treatment. Three pediatric patients (10.7% of the cohort) were included in the study (one patient aged 12, two aged 15). In total, 16 patients presented with genotype F508del with homozygosity and the remaining 12 patients had the genotype F508del with heterozygosity. The type of CFTR modulator therapy used before ETI was lumacaftor/ivacaftor in 12 patients, tezacaftor/ivacaftor in 2 patients; the remaining 14 patients had not taken any modulator therapy prior to ETI. We analyzed the insulin regimen of each patient: 16 patients used only basal insulin, whereas 12 patients followed the basal-bolus scheme. The demographic and clinical characteristics are shown in Table 1.

The main results are shown in detail in Table 2.

The TDD (IU) significantly decreased at T_3mo_ and T_6mo_, but not at T_12mo_, whereas HbA1c significantly decreased at all time points. The results of the chloride sweat test (mmol/L) also decreased significantly, as expected. The number of hospitalizations (n) and pulmonary exacerbations significantly decreased over the study period. All pulmonary function parameters improved significantly at 3, 6 and 12 months, particularly the parameter FEV1%. The patients’ nutritional status significantly improved over the observational period; the BMI (kg/m^2^) increased significantly. The psychological status and quality of life of our patients were assessed via CFQ-R and appeared to improve significantly in the first year of ETI treatment.

The results of the subanalysis based on diverse genotypes are illustrated in detail in the Supplementary Material.

The total daily dose of insulin (IU) in homozygous F508del patients only significantly decreased at T0 vs. T_6mo_ and HbA1c (%) only significantly decreased at T0 vs. T_3mo_. In heterozygous F508del patients, HbA1c decreased significantly at T0 vs. T_6mo_ and T0 vs. T_12mo_. The BMI improved significantly in both groups, except for T_12mo_ in the homozygous F508del group. As for pulmonary function, all spirometry parameters significantly increased at 3, 6 and 12 months. Patients with heterozygous F508del showed significantly higher FEV1 (mL) at 3 months and 6 months.

## 4. Discussion

To the best of our knowledge, this is the first retrospective observational study to demonstrate both an improvement in glycemic control (by means of HbA1c) and reduction in insulin needs after ETI therapy in a cohort of pediatric and adult insulin-dependent patients with CFRD.

Our results regarding decreased HbA1c values are concordant with the only other study that demonstrates glycemic control improvement in patients with CFRD under ETI treatment [9]. Evidence supporting our findings in patients undergoing CFTR modulator therapy up until now has been contrasting or inconclusive, highlighting only favorable trends in glycemic control in patients with CFRD or impaired glucose intolerance [15,16] or no improvement at all [17]. Volkova et al. suggest that the prevalence of CFRD was lower in patients who received only ivacaftor, therefore underlining the efficacy of the sole use of a single CFTR modulator. This was also noted by Tsabari et al. who examined a cohort that was only administered ivacaftor [15,16]. Our results highlight the innovative amelioration in glycemic control while using the triple combined modulator therapy.

Many studies have demonstrated ETI’s superiority compared to other modulators for various CF measures such as FEV1, sweat chloride levels, BMI and pulmonary exacerbations [18,19]. Clear glycemic and metabolic outcomes regarding ETI treatment are less studied and yet to be demonstrated. Scully et al. were among the first to demonstrate the potential improvements in glycemic control following ETI treatment, but chose a study period of 3–11 months for a mixed population (patients with and without CFRD) [7]. These results stand in contrast to those reported by Crow et al. who demonstrated no statistically significant changes in their measures of glycemia and HbA1c, or in basal insulin requirements after 6 months of ETI treatment [20]. This stands in contrast to our study in which both HbA1c and insulin requirements were statistically lower after ETI treatment. The physiopathological mechanism behind the amelioration of glycemic control is still unclear and the explanation is likely to be multifactorial, including better intestinal absorption and nutritional status, decreased inflammatory state, enhanced beta cell function and improved insulin sensitivity [9].

Compared to the baseline, we observed a reduction in insulin requirement at 3 months and 6 months, but not at 12 months of ETI therapy. Sustained reductions in the total daily insulin doses at 12 months may not have been evident due to the patients’ increased appetite or improved adherence to CFRD treatment, leading to higher insulin doses per kg. No patients discontinued insulin therapy after initiating ETI treatment. More studies are needed to confirm the potentiated effect of ETI treatment in the first few months that could explain the reduction at the beginning of the treatment. HbA1c levels at all three times were significantly lower than at T0. Further studies and longer periods of observation are necessary to better understand this phenomenon.

The cohort’s pulmonary function improved and all parameters assessing clinical and pulmonary improvements, such as spirometry and number of pulmonary exacerbations and hospitalizations, significantly improved in agreement with previously published studies, as well as nutritional status and BMI [21,22,23,24,25,26].

Because depression and anxiety are frequently diagnosed in patients with CF and associated with a lower quality of life and a lower adherence to airway clearance treatment, we concluded that patients’ mental health status is an important outcome. Indeed, mental health appeared to improve significantly in our patients after beginning ETI treatment according to CFQ-R data, in agreement with other studies [27,28].

We subanalyzed our data based on two different genotypes, F508del with homozygosity and with heterozygosity. Pulmonary function improved constantly and significantly in patients who had F508del with homozygosity, while heterozygous F508del patients showed only a minor improvement. These data are likely explained by the fact that patients with heterozygous F508del have a better FEV1 and a smaller margin of improvement in our cohort of patients. The significant increase in BMI our results are in line with other studies in which weight gain appears to be higher in patients with F508del with heterozygosity [29,30]. As for insulin requirements and glycemic control, our results appear contrasting between the two groups, where heterozygous F508del patients did not show any reduction in insulin requirements after one year of treatment, despite a significant reduction in HbA1c values at 6 and 12 months. On the other hand, homozygous F508del patients significantly decreased the amount of insulin required, which was only significant at T_6mo_, accompanied by a smaller decrease in HbA1c values at T_3mo_.

This study has some limitations including its retrospective nature and the lack of visits of some patients at 3–6–12 months post-ETI treatment initiation, as well as the small sample size with only a short follow-up period.

## 5. Conclusions

In conclusion, our study demonstrated that ETI therapy both significantly improved glycemic control and significantly decreased insulin requirement in a cohort of adult and pediatric patients with CFRD. Further studies with a larger population and a longer follow-up period are needed to better understand the trends in insulin requirements in patients with CFRD on ETI treatment and to appropriately adjust insulin therapy during their follow-up. In future studies, the use of continuous glucose monitoring in our patients affected by CFRD would allow us to consolidate our data.

## Figures and Tables

**Table 1:** Demographic and clinical characteristics.

Total = 28 Patients		
	Mean ± SD	Median (min, max)
Age (years)	33.5 ± 13.26	33.0 (12, 59)
Age at diagnosis CFRD (years)	24.9 ± 9.65	23.66 (9, 44)
Duration of insulin (years)	7.77 (±7.76)	4.67 (0.56, 32.19)
	**Frequency (N)**	**Percentage (%)**
**Sex**		
*Female*	12	42.9
*Male*	16	57.1
**Genotype**		
*F508del/F508del (homozygous)*	16	57.1
*F508del/other (heterozygous)*	12	42.9
**Previous use of CFTR modulators**		
*None*	14	50.0
*Lumacaftor/ivacaftor*	12	42.9
*Tezacaftor/ivacaftor*	2	7.1
**Insulin regimen**		
*Once daily basal insulin*	16	57.1
*Basal-bolus*	12	42.9

**Table 2:** Pulmonary function and metabolic changes in patients affected by cystic fibrosis-related diabetes before and after treatment with elexacaftor/tezacaftor/ivacaftor. Pre-T0: 1 year before; T0: treatment initiation; T_3mo_: 3 months after; T_6mo_: 6 months after; T_12mo_ 1 year after. FEV1: forced expiratory volume in the first second; FVC forced vital capacity; HBA1c: glycated hemoglobin; CFQR: cystic fibrosis questionnaire revised.

	N	Pre-T0	N	T0	T_3mo_	T0 vs. T_3mo_	N	T_6mo_	T0 vs. T_6mo_	N	T_12mo_	T0 vs. T_12mo_
Hospitalizations (n)	28	0.86 ± 0.89								28	0.32 ± 0.48	**0.003**
Pulmonary exacerbations (n)	28	1.64 ± 1.59								28	0.46 ± 0.79	**0.002**
Six-minute walking test (meters)	19	574.18 ± 68.04								19	572.75 ± 92.99	0.71
Chloride sweat test (mmol/L)	15	118.53 ± 22.21								15	49.93 ± 17.81	**0.001**
Body mass index (kg/m^2^)			26	21.72 ± 2.88	22.53 ± 3.11	**0.0001**	26	22.48 ± 3.02	**0.0001**	25	22.47 ± 3.46	**0.006**
FEV1 (liters/1 s)			27	2420.0 ± 1038.4	2782.2 ± 946.6	**0.0001**	27	2774.8 ± 991.7	**0.0001**	25	2771.6 ± 967.1	**0.001**
FVC (liters)			27	3267.4 ± 1043.3	3640.0 ± 899.1	**0.001**	27	3694.8 ± 959.8	**0.0001**	25	3671.5 ± 938.7	**0.001**
FEV1%			27	75.74 ± 29.21	85.44 ± 23.70	**0.001**	27	85.37 ± 25.40	**0.002**	25	85.68 ± 23.12	**0.01**
Total daily insulin dose (UI/kg)			27	0.26 ± 0.22	0.18 ± 0.18	**0.01**	25	0.21 ± 0.20	**0.04**	23	0.21 ± 0.22	0.10
HbA1c (%)			23	6.17 ± 0.63	5.94 ± 0.58	**0.001**	26	5.89 ± 0.59	**0.004**	23	5.94 ± 0.60	**0.006**
CFQ-R (n)			26	64.63 ± 7.59			26	71.74 ± 4.12	**0.0001**	15	70.13 ± 4.84	**0.01**

## Data Availability

The datasets generated during and/or analyzed in the current study are available from the corresponding author upon reasonable request.

## Supplementary Materials

The following supporting information can be downloaded at https://www.mdpi.com/article/10.3390/life14101309/s1; Table S1: Pulmonary function and metabolic changes in patients affected by cystic fibrosis-related diabetes with genotype F508del/F508del before and after treatment with elexacaftor/tezacaftor/ivacaftor. Pre-T0: 1 year before; T0: treatment initiation; T1: 3 months after; T2: 6 months after; T3 1 year after. FEV1: forced expiratory volume in the first second; FVC forced vital capacity; HBA1c: glycated hemoglobin. Table S2: Pulmonary function and metabolic changes in patients affected by cystic fibrosis-related diabetes with genotype F508del/any other CFTR mutation before and after treatment with elexacaftor/tezacaftor/ivacaftor. Pre-T0: 1 year before; T0: treatment initiation; T1: 3 months after; T2: 6 months after; T3 1 year after. FEV1: forced expiratory volume in the first second; FVC forced vital capacity; HBA1c: glycated hemoglobin.
