# Supplementary material for: One-Year Effect of Elexacaftor/Tezacaftor/Ivacaftor Therapy on HbA1c Levels and Insulin Requirement in Patients with Insulin-Dependent Cystic Fibrosis-Related Diabetes: A Retrospective Observational Study
Source: Life (Basel). 2024 Oct 16;14(10):1309. doi: 10.3390/life14101309 (PMC11509452; doi:10.3390/life14101309)
Supplement: Supplementary file 1 [file life-14-01309-s001.zip › life-3209268-supplementary.pdf]

Supplementary Material

Table S1: Pulmonary function and metabolic changes in patients affected by cystic fibrosis related diabetes with genotype F508del/F508del before and after treatment with elexacaftor/tezacaftor/ivacaftor. Pre-T0: 1 year before; T0: treatment initiation; T_{3mo}: 3 months after; T_{6mo}: 6 months after; T_{12mo} 1 year after. FEV1: forced expiratory volume in the first second; FVC forced vital capacity; HBA1c: glycated hemoglobin; CFQR: cystic fibrosis questionnaire revised.

	N	Pre-T0	N	T0	T_{3mo}	T0 vs T_{3mo}	N	T_{6mo}	T0 vs T_{6mo}	N	T_{12mo}	T0 vs T_{12mo}
Hospitalizations (n)	16	0.75±0.58								16	0.31±0.48	**0.02**
Pulmonary exacerbations (n)	16	1.44±1.59								16	0.50±0.89	0.08
Six minute walking test (metres)	11	560.67±80.87								11	542.95±96.99	0.96
Chloride sweat test (mmol/L)	6	114.83±13.73								6	52.00±22.64	**0.03**
Body mass index (kg/m^2)			15	20.91±2.47	21.49±2.43	**0.01**	14	21.44±2.46	**0.01**	14	21.20±2.90	0.21
FEV1 (litres/1 sec)			16	2326.9±871.8	2696.9±944.15	**0.0001**	15	2720.7±953.11	**0.001**	14	2803.57±937.4	**0.001**
FVC (litres)			16	3110.0±936.2	3463.1±919.2	**0.004**	15	3524.0±920.3	**0.006**	14	3586.3±936.4	**0.004**
FEV1%			16	72.00±24.27	83.19±24.46	**0.001**	15	84.47±24.41	**0.001**	14	87.21±21.42	**0.001**
Total daily insulin dose (UI/kg)			14	0.29±0.21	0.20±0.20	0.06	13	0.19±0.19	**0.05**	12	0.21±0.21	0.21
HbA1c (%)			13	6.25±0.75	6.01±0.59	**0.005**	15	6.09±0.64	0.29	12	6.09±0.66	0.19

.

Table S2: Pulmonary function and metabolic changes in patients affected by cystic fibrosis related diabetes with genotype F508del/any other CFTR mutation before and after treatment with elexacaftor/tezacaftor/ivacaftor. Pre-T0: 1 year before; T0: treatment initiation; T_{3mo}: 3 months after; T_{6mo}: 6 months after; T_{12mo} 1 year after. FEV1: forced expiratory volume in the first second; FVC forced vital capacity; HBA1c: glycated hemoglobin; CFQR: cystic fibrosis questionnaire revised.

	**N**	**Pre-T0**	**N**	**T0**	**T_{3mo}**	**T0 vs T_{3mo}**	**N**	**T_{6mo}**	**T0 vs T_{6mo}**	**N**	**T_{12mo}**	**T0 vs T_{12mo}**
Hospitalizations (n)	12	1.00±1.21								12	0.33±0.49	0.07
Pulmonary exacerbations (n)	12	1.92±1.62								12	0.42±0. 67	**0.01**
Six minute walking test (metres)	8	592.75±43.35								8	613.71±74.07	0.57
Chloride sweat test (mmol/L)	9	121.00±26.99								9	48.56±15.14	**0.008**
Body mass index (kg/m^2)			11	22.82±3.15	23.95±3.48	**0.004**	12	23.69±3.25	**0.01**	11	24.10±3.54	**0.005**
FEV1 (litres/1 sec)			11	2555.5±1276.5	2906.4±981.87	**0.04**	12	2842.5±1076.7	**0.04**	11	2730.9±1048.4	0.18
FVC (litres)			11	3496.4±1190.9	3897.3±843.6	0.09	12	3908.3±1005.0	**0.03**	11	3780.0±975.6	0.11
FEV1%			11	81.18±35.78	88.73±23.29	0.19	12	86.50±27.64	0.33	11	83.73±26.07	0.72
Total daily insulin dose (UI/kg)			11	0.22±0.23	0.15±0.17	0.12	12	0.22±0.22	0.40	11	0.22±0.25	0.26
HbA1c (%)			10	6.07±0.45	5.84±0.57	0.06	11	5.62±0.40	**0.004**	11	5.77±0.51	**0.02**
